# A Common Variant at the 3'untranslated Region of the CCL7 Gene (rs17735770) Is Associated With Decreased Susceptibility to Coronary Heart Disease

**DOI:** 10.3389/fcvm.2022.908070

**Published:** 2022-05-31

**Authors:** José María Medina-Gil, Ana Pérez-García, Pedro Saavedra-Santana, Asunción Díaz-Carrasco, Efrén Martínez-Quintana, Fayna Rodríguez-González, Cristina M. Ramírez, Marta Riaño, Paloma Garay-Sánchez, Antonio Tugores

**Affiliations:** ^1^Servicio de Cardiología, Complejo Hospitalario Universitario Insular Materno-Infantil, Las Palmas de Gran Canaria, Spain; ^2^IMDEA Research Institute of Food and Health Sciences, Madrid, Spain; ^3^Departamento de Matemáticas, Universidad de Las Palmas de Gran Canaria, Las Palmas de Gran Canaria, Spain; ^4^SECUGEN SL, Madrid, Spain; ^5^Servicio de Oftalmología, Hospital Universitario Gran Canaria Doctor Negrín, Las Palmas de Gran Canaria, Spain; ^6^Servicio de Bioquímica Clínica y Análisis Clínicos, Complejo Hospitalario Universitario Insular Materno-Infantil, Las Palmas de Gran Canaria, Spain; ^7^Unidad de Investigación, Complejo Hospitalario Universitario Insular Materno-Infantil, Las Palmas de Gran Canaria, Spain

**Keywords:** chemokine, CCL7, genetics, microRNA (miRNA), cardiovascular disease

## Abstract

Monocytes participate in the development of atherosclerosis through the action of cytokines and other inflammatory mediators. Among them, CCR2 and its ligands, CCL2 and CCL7 play an important role, so the main objective of this work was to determine whether genetic variants affecting their activity were associated with cardiovascular disease. A cohort of 519 patients that have suffered coronary events was analyzed under a propensity score-matching protocol selecting a homogeneous set of cases and controls, according to age, sex, smoking status, dyslipidemia, arterial hypertension and type 2 diabetes as risk factors. While dyslipidemia and arterial hypertension were more prevalent among patients with angina pectoris, current smoking status and elevated inflammatory markers, including total leukocyte and monocyte counts, were more likely associated with acute coronary events. Propensity score matching analysis, performed to eliminate the influence of these risk factors and highlight genetic modifiers, revealed that a single nucleotide variant, rs17735770 at the 3'untranslated region of the CCL7 gene transcript, was associated with decreased cardiovascular risk in a group represented mostly by men, with an average age of 57, and without significant differences in traditional risk factors. Furthermore, the presence of this variant altered the local mRNA structure encompassing a binding site for miR-23ab, resulting in increased translation of a reporter gene in a miR23 independent fashion. The rs17735770 genetic variant led to increased expression of CCL7, a potential antagonist of CCR2 at inflammatory sites, where it could play a meaningful role during the evolution of atherosclerosis.

## Introduction

Beyond classical cardiovascular risk factors, inflammation appears as a major player in the formation, evolution and maturation of atheroma plaques in the endothelium (1, 2). Monocytes play a major role from start in this process, adhering to the endothelium and infiltrating into inflammatory sites in response to chemoatractive chemokines, becoming macrophages and, later, foam cells which are the origin of the intimal fat strike (3). After death, foam cells contribute to the formation of the necrotic core of the plaque and, by releasing metalloproteinases and cathepsins, destabilize the plaque by thinning the fibrous outer capsular layer which, upon breakage and tissue factor release, favors coronary thrombosis and the occurrence of an acute coronary event (1–3).

Chemokines and their receptors are important mediators of inflammation and tissue morphogenesis, including hematopoiesis and angiogenesis, through the induction of chemotaxis, proliferation and apoptosis of many cell types (4). In particular the C-C type chemokine receptor type 2 (CCR2) and its ligand CCL2, and later CX3CR1 and CCR5 have been associated with the recruitment of monocytes to inflammatory sites (5–8).

Mice deficient in the *CCR2* gene show defects in monocyte migration, reduced leukocyte adhesion, and monocyte recruitment at the plaques (9–11) and rescue the development of atherosclerotic lesions under a high fat diet, when crossed to hyperlipidemic mice, deficient in ApoE (12, 13). Accordingly, mice deficient in *CCL2* are more resistant to the development of atherosclerosis in response to a high cholesterol diet than their wild type littermates (14, 15).

While CCL2 is an exclusive CCR2 ligand, CCL7 may also bind to other chemokine receptors present in a variety of cell types (4). At inflammatory sites, where there is a presence of metalloproteinases such as gelatinases (MMP-2 y MMP-9) and MMP-7, the CCL7 molecule may be proteolyzed to form a competitive CCR2 antagonist that may modulate its response (16).

Given the importance of the CCR2 receptor in the development of atherosclerosis in mice, some efforts have been directed toward the identification of genetic variants that may modulate its activity in humans. The frequent polymorphic variant p.Val64Ile (SNP Id: rs1799864) has been associated with increased risk of myocardial infarction and heart failure (17), but not always (18–21), and is also associated with decreased coronary calcification (22). Likewise, the association of variations in the ligand CCL2 with cardiovascular disease is also controversial (23–25). Therefore, the possibility that genetic variants in this receptor-ligand system could influence the development of cardiovascular disease in our patients was explored.

## Materials and Methods

### Subjects

All participants gave written informed consent before being included in the study, which was also approved by the Ethics Committee of our institution. The study included 519 patients, between 25 and 90 years old, that presented with a coronary event and required hospital admission, and a set of 760 controls (26). Excluded from the study were patients without a clear coronary event diagnosis, those with cancer, fever, or any known inflammatory disease, and those that did not wish to participate. In-hospital data at admission included: anthropomorphic and demographic data, analytical data and the type of acute coronary event: stable angina, unstable angina, non-ST (NSTEMI) and ST-elevation myocardial infarction (STEMI) (27).

### Genetic Analysis

Genomic DNA was extracted from white blood cells with the Puregene kit (QUIAGEN, Hilden, Germany). An initial screen for the detection of genetic variants in the *CCR2, CCL2* and *CCL7* genes was performed in 70 cases and 30 healthy controls by heterozygous double pass Sanger sequencing of amplified DNA fragments containing coding exons and flanking sequences. All primers used for polymerase chain reaction (PCR) are shown in a Supplementary File.

Sequences were analyzed with the 4Peaks software (Nucleobytes Inc., The Netherlands), and aligned against reference sequences using BLAST (28) to identify variants, that were later searched in the dbSNP database (29). Genotyping of the rs17735770 variant involved the design of an RFLP (restriction fragment length polymorphism) assay, as the wild type to variant transition generates a novel *SnaBI* restriction site. Primers used for PCR (see Supplementary Methods for sequences) were designed with the PrimerBLAST tool (30). Genotyping was performed on 771 controls and 519 cases by using PCR followed by fragment electrophoretic analysis essentially as described (31).

### Bioinformatic Analysis

Species-specific searches to detect alignment of the *CCL7* gene 3' UTR region in mammals was performed with BLAST (28). In order to detect predicted miRNA binding motifs in the region containing the rs17735770 variant, the TargetScanHuman® v. 5.1 application (32) was used as implemented on the UCSC Genome Browser (33). The algorithms RNAfold and RNAcofold were used on the Vienna RNA websuite (34).

### 3'UTR Luciferase Reporter Assays

cDNA fragments corresponding to the entire 3'UTR of the human CCL7 gene were amplified from human genomic DNA by PCR with Phusion Hot Start II DNA Polymerase (Thermo Fisher Scientific, Waltham, MA) using primers flanked by *XhoI* (5' forward primer) and *NotI* (3' reverse primer) overhangs (sequences in Supplementary Methods). The resulting PCR products were directionally cloned into the *XhoI* and *NotI* sites of the psiCHECK-2 vector (Promega Biotech, Madison, WI), downstream of the *Renilla* luciferase (hRluc) ORF. The vector also contains a constitutively expressed humanized *Photynus* luciferase (hluc) gene, which is used to normalize transfections. The rs17735770 variant and a disrupting point mutation in the seed region of the predicted miR-23a-3p site (AATGTGAA to AATCAGGA) within the *CCL7* 3'UTR were generated by using QuikChange Multi Site Directed-Mutagenesis Kit (Agilent Technologies Inc, Wood Dale, IL) according to the manufacturer's protocol (primers sequences in Supplementary Methods). All constructs were checked by Sanger sequencing of individual clones.

HEK and COS-7 cells were plated into 12-well plates and co-transfected with 1 μg of the indicated 3'UTR luciferase reporter vectors and 40nM of the miR-23a-3p mimic or nontargeting control mimic (CM) sequence (Horizon Discovery, formerly Dharmacon, Lafayette, CO) utilizing Lipofectamine 2000 (Invitrogen-Thermo Fisher Scientific). In the case of THP-1, cells were treated with 100 nM phorbol myristate acetate (61 mg/ml PMA) for 24 h before transfection. Luciferase activity was measured 24 h after transfection using the Dual-Glo Luciferase Assay System (Promega). *Renilla* luciferase activity was normalized to the corresponding *Photynus* luciferase activity and plotted as a percentage of the control (cells co-transfected with the corresponding concentration of control mimic). At least three independent experiments were performed in triplicates.

### Statistical Analysis

Categorical variables were expressed as frequencies and percentages and continuous as means and standard deviations (SD). The percentages were compared, as appropriate, using the Chi-square (χ^2^) test or the exact Fisher test and the means by the *t*-test. Multiple comparisons were carried out, as appropriate, using Scheffe's method or a non-parametric approach (35).

In order to determine the association between the presence of coronary heart disease (CHD) and the rs17735770 variant (T → C) at the *CCL7* gene, a similar control was selected for each case (matching), based on a propensity score, defined as the conditional probability of suffering CHD given a set of covariates obtained for each individual using logistic regression, in which the outcome variable was CHD. The covariates included in the model were selected using the best subset regression procedure together with and Akaike Information Criterion (AIC) (36).

A 1-to-1 matched analysis without replacement on the basis of the estimated propensity score of each patient was performed. The caliper chosen was 0.3. After propensity score matching, baseline characteristics were compared with the McNemar tests for binary variables and the *t*-tests or Wilcoxon test, as appropriate, for continuous variables, both for paired data. In addition, we assessed the success of propensity score matching to balance covariates in the two groups using standardized differences. Standardized differences of < 10% support the assumption of balance between the 2 groups (37). Statistical significance was set at *p* < 0.05. Data were analyzed using the R package, version 3.3.1 (38).

## Results

### Association of Cardiovascular Risk Factors With Disease

A total of 519 cases presenting with a cardiovascular event were compared with a group of 760 controls. Both groups showed strong differences in age (61.3 in cases vs. 48.9 years in controls, *p* < 0.001), sex distribution (72.5% were male in the cases group vs. 45.4% in controls, *p* < 0.001), smoking (61.9 vs. 39.0%, *p* < 0.001), arterial hypertension (65.7 vs. 22.9%; *p* < 0.001), diabetes mellitus type 2 (T2DM) (40.3 vs. 27.1%; *p* < 0.001) and dyslipidemia (53.2 vs. 33.4%; *p* < 0.001) (Table 1).

**Table 1:** Cardiovascular risk factors among patients and controls.

	***Coronary Heart Disease***		
	**No** ***N* = 760**	**Yes** ***N* = 519**	***P***
Age (years)	48.9 ± 12.1	61.3 ± 12.2	<0.001
Sex male (%)	345 (45.4)	376 (72.5)	<0.001
Body Mass Index (Kg/m^2^)	28.3 ± 5.1	29.5 ± 5.4	0.088
Smoking^a^, *n* (%)			<0.001
No	460 (61.1)	198 (38.2)	
Ex-smoker	112 (14.5)	106 (20.4)	
Current	188 (24.4)	215 (41.4)	
Smoker (current + ex smokers)	296 (39)	321 (61.9)	<0.001
Arterial hypertension^b^	174 (22.9)	341 (65.7)	<0.001
Type-2 diabetes mellitus^c^	206 (27.1)	209 (40.3)	<0.001
Triglycerides (mg/dl)	106 (76; 149)	131 (100; 169)	<0.001
Cholesterol (mg/dl)			
Total	212.1 ± 40.0	163.9 ± 44.0	<0.001
LDL	132.4 ± 35.9	93.3 ± 34.6	<0.001
HDL	53 (46, 61)	36 (31, 43)	<0.001
Dyslipidemia^d^	254 (33.4)	276 (53.2)	<0.001
Statins^e^	123 (16.2)	481 (95.1)	<0.001
Leucocytes ( ×10 exp 3/ml)	7.50 (6.00; 8.70)	8.60 (7.00; 10.40)	0.009

a *Patients were considered current smokers if they were actively smoking at the time of the event or had smoked regularly within a 2 year period before the event and ex-smokers if they had smoked before but not during the last 2 years*.

b *Arterial hypertension (AHT) was considered when systolic blood pressure > 140 mmHg or diastolic blood pressure > 90 mmHg, or when the patient was already receiving medication for hypertension*.

c *Diabetes mellitus: when fasting blood glucose levels > 126 mg/dl or patient treated with oral anti-diabetic agents or insulin*.

d *Dyslipidemia (DLP) was considered if total cholesterol levels > 240 mg/dl. LDL>160 mg/dl or if patient was receiving lipid-lowering therapy*.

e *Statins used were mainly atorvastatin 20–80 mg/24 h, rosuvastatin 10–20 mg/24 h, or simvastatin 20–40 mg/24 h. Data are means ± SD and frequencies (%)*.

Patients were also classified according to the type of event. Current smokers were more frequent among the STEMI patients. While body mass index differences were not significant among all four groups, diabetes and dyslipidemia appeared associated with angina rather than with acute events. Total leukocyte and platelet counts, along with levels of C reactive protein (CRP) and fibrinogen, increased in the NSTEMI group, showing maximal values in the STEMI patients (Table 2).

**Table 2:** Characteristics of patients according to the type of event.

	**Angina**		**AMI**		
	**Stable** ***N* = 27**	**Unstable** ***N* = 81**	**NSTEMI** ***N* = 214**	**STEMI** ***N* = 197**	***P***
Age, years	61.7 ± 10.5^**ab**^	61.0 ± 10.2^**ab**^	63.7 ± 12.1^**a**^	58.8 ± 12.8^**b**^	<0.001
Male, %	23 (85.2)	55 (67.9)	153 (71.5)	145 (73.6)	0.353
Body mass index	28.7	31.1 ± 4.5	28.8 ± 4.2	29.4 ± 6.5	0.764
Smoker	12 (44.4)	51 (63.0)	131 (61.2)	127 (64.5)	0.249
Smoking, *n* (%)					0.009
No	15 (55.6)	30 (37.0)	83 (38.8)	20 (35.5)	
Ex-smoker	6 (22.2)	23 (28.4)	49 (22.9)	28 (14.2)	
Current	6 (22.2)	28 (34.6)	82 (38.3)	99 (50.3)	
Arterial hypertension, *n* (%)	22 (81.5)^**a**^	60 (74.1)^**a**^	155 (72.4)^**a**^	104 (52.8)^**b**^	<0.001
Type 2 diabetes mellitus, *n* (%)	13 (48.1)^**a**^	34 (42.0)^**a**^	105 (49.1)^**a**^	57 (28.9)^**b**^	<0.001
Dyslipidemia	22 (81.5)^**a**^	53 (65.4)^**a**^	122 (57.0)^**a**^	79 (40.1)^**b**^	<0.001
Leukocytes ( ×10^3^/ml)	7.4 (6.1–8.3)^**a**^	7.8 (5.9–9.9)^**ab**^	8.4 (7.2–10.0)^**b**^	9.6 (7.5–11.0)^**c**^	<0.001
Platelets ( ×10^3^/ml)	202 (168–246)^**a**^	221 (188–256)^**a**^	225 (186–277)^**a**^	255 (204–314)^**b**^	<0.001
Fibrinogen, gr/L	4.1 (3.5–5.0)^**ab**^	3.8 (3.5–4.2)^**a**^	4.0 (3.5–4.5)^**a**^	4.8 (4.2–5.4)^**b**^	<0.001
CRP, mg/L	0.6 (0.2–2.1)^**a**^	0.5 (0.2–1.4)^**a**^	1.4 (0.5–3.6)^**a**^	2.9 (1.5–7.0)^**b**^	<0.001

*Data shown are frequencies (%), means ± SD or medians (IQR) at 95% CI. Different superscripts indicate significant differences (p < 0.05). See legend of Table 1 for details. AMI, Acute myocardial infarction; NSTEMI, non-ST-elevation myocardial infarction; STEMI, ST-elevation myocardial infarction; CRP, C Reactive Protein*.

### Association of Genetic Variants in the CCR2, CCL2, and CCL7 Genes With Coronary Heart Disease

The significant elevation of leukocytes and inflammatory markers in acute events emphasized the association of inflammation with coronary heart disease in this cohort. Given the potential role of the CCR2 chemokine receptor in progression of atherosclerosis, the hypothesis that genetic variants that could affect function of the receptor and its ligands could represent a risk factor in this setting was explored. Seventy cases and 30 healthy controls were selected to sequence all coding and flanking sequences of the *CCR2, CCL2* and *CCL7* genes. Assuming a dominant model, this approach would examine 70 “disease” and 130 “control” alleles.

A total of 16 genetic variants were found in *CCR2*, 2 in *CCL2* and 4 in *CCL7*. Out of these, there was a single common non synonymous variant (rs1799864) affecting *CCR2*. Two novel variants were also found on this gene (3:46354176; 3:46354213), and a third one, an indel (rs372263390), had no frequency data available. Among all other variants found, only one appeared to be underrepresented in cases (2/70) vs. controls (6/30), rs17735770, located at the 3'untranslated region (UTR) of the *CCL7* gene (see Supplementary Table).

Before attempting an analysis of the association with the rs17735770 variant with disease, and because of significant differences observed between affected and controls, a matching based on the propensity score was carried out. First, all related variants associated with disease were identified through a multivariant logistic regression analysis, using the complete enumeration algorithm and Akaike Information Criterion (AIC). Table 3 summarizes the logistic model in which was based the propensity score. The covariates that showed independent association with the coronary heart disease (CHD) variable were age (*p* < 0.001), sex (male) (*p* < 0.001), smoker status (*p* < 0.001), dyslipidemia (*p* = 0.053), arterial hypertension (*p* < 0.001) and type 2 diabetes mellitus (*p* = 0.025). From the 519 patients with CHD, a subset of 305 was obtained, which resulted in a set of homogeneous pairs of cases and controls in terms of the variables considered.

**Table 3:** Multivariate logistic regression for *Coronary Heart Disease*.

	**Coefficient (SE)**	**P-value**	**OR (95% CI)**
Intercept	−6.171 (0.408)	<0.001	–
Age, per year	0.072 (0.007)	<0.001	1.075 (1.061; 1.089)
Sex male	0.940 (0.163)	<0.001	2.560 (1.861; 3.523)
Smoker	1.204 (0.168)	<0.001	3.332 (2.399; 4.630)
Arterial hypertension	1.445 (0.162)	<0.001	4.242 (3.088; 5.828)
Dyslipidemia	0.286 (0.148)	0.053	1.331 (0.996; 1.778)
Type-2 diabetes mellitus	−0.367 (0.163)	0.025	0.693 (0.503; 0.954)

*Selection of variables was carried out using the best subset regression procedure together with the Akaike Information Criterion*.

After propensity score matching of where the related variables were introduced, 305 patients were selected for which a homogenous pair was found for the referred variables using a caliper of 0.5. As shown (Table 4) a set of homogeneous pairs of cases and controls in terms of the variables considered was selected, with similar characteristics in patients with CHD vs. controls: mostly by men, with an average age of 57, and without significant differences in traditional risk factors such as smoking, dyslipidemia, arterial hypertension or type 2 diabetes, as all standardized differences diverged < 10%, supporting the assumption of a balance between both groups. After pairing, a conditional logistic regression analysis revealed that carrying the C variant at the rs17735770 *CCL7* gene variant appeared as a protection factor for CHD (OR = 0.333, 95% CI = 0.157–0.709) (Table 5).

**Table 4:** Patient characteristics of the study cohorts after Propensity score matching.

	***Coronary Heart Disease***			
.	**No** ***N* = 305**	**Yes** ***N* = 305**	***P***	**% Standardized** difference^*^
Age (years)	57.4 ± 10.9	58.6 ± 12.2	0.049	9.58
Sex male	190 (62.9)	189 (62.6)	0.924	−0.68
Smoker	171 (56.6)	161 (53.3)	0.220	−6.63
Dyslipidemia	150 (49.7)	154 (51.0)	0.746	2.65
Arterial hypertension	140 (46.4)	150 (49.7)	0.282	6.61
Type-2 diabetes mellitus	112 (37.1)	126 (41.7)	0.167	9.39

The chosen caliper was 0.4 (*). Data are means ± SD and frequencies (%). Note that all standardized differences were < 10%.

**Table 5:** Conditional logistic regression for the CHD variable.

	**Controls (302)**	**CHD (302)**	***P***	**Odd-Ratio (95% CI)**
CCL7			0.009	–
TT	269 (89.1)	287 (95.0)		1 (Reference)
CT or CC	33 (10.9)	15 (5.0)		0.419 (0.219–0.801)

*Evaluation of the presence of the rs17735770 variant (T > C) after matching by the propensity score*.

### Predicted Functional Consequence of the rs17735770 Variant

**Figure 1:**
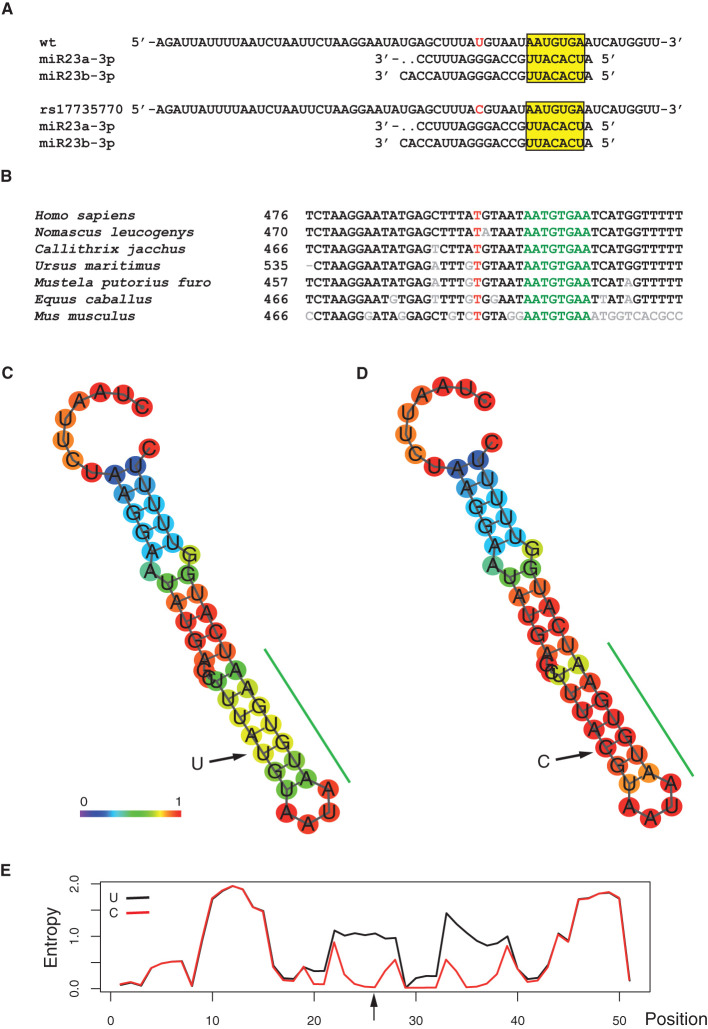
Predicted functional significance of the of the rs1773577 variant. **(A)** Context sequence of the rs1773577 variant aligned with mammalian species. Numbers indicate the position on the mRNA. The position affected by the rs1773577 variant is red, while the miR28ab binding site predicted by the TargetScan software.is shown in green. Non-conserved positions are indicated in gray. Reference sequences shown are: NM_006273.3 (*Homo sapiens*); XM_003278361.3 (*Nomascus leucogenys*); XM_002748371.3 (*Callithrix jacchus*); XM_008695392.1 (*Ursus maritimus*); XM_004747090.2 (*Mustela putorius furo*); XM_005597581.1 (*Equus caballus*); NM_013654.3 (*Mus musculus*). **(B)** Alignment of the predicted miR23 binding sequence with the corresponding mature miR23a and b. **(C–E)** RNAfold algorithm analysis of the effect of the rs1773577 variant in its RNA structural context. Panel **(C)** shows a minimum free energy (MFE) drawing of a local hairpin for the wt (T) allele pairing prediction, while panel **(D)** shows the same prediction for the variant **(C)**. Color coding indicates base-pair probabilities as shown on the figure, at the bottom of panel **(C)**. The green bar on the side of the predicted structures indicates the *miR23ab* predicted binding site. **(E)** Graphical representation of the level of entropy for each position along both structures, as indicated.

The TargetScanHuman® v. 5.1 application (32), which uses all known miRNA heptanucleotide binding sequences present in the 3'UTR sequences from all known mammalian genes, was used to detect that the rs17735770 variant was in close proximity (6 base pairs away) of a high score (98/100) 8-mer binding site, for the microRNAs miR-23a and b, generically known as miR-23ab (Figure 1A). The *CCL7* gene, as revealed by similarity BLAST (28) searches, was present, exclusively, in mammals and, despite being present in the least conserved 3'UTR of the gene, the immediate vicinity of the variant is highly conserved among different mammalian species (Figure 1B). Indeed, this consensus octamer binding site, at the 3'UTR of the *CCL7* gene has been suggested to be recognized by miR-23 and regulate CCL7 expression in mice (39).

As the rs17735770 variant lied within the 3ÚTR of the CCL7 gene, in close proximity to a miR-23ab binding site, the possibility that this variant could affect the local RNA structure was investigated *in silico* using the RNAfold algorithm (34), which calculates the minimum free energy (MFE) structure for every particular sequence. Therefore, the effect of a single nucleotide variant on the secondary structure of an RNA molecule can be evaluated by comparing the resulting structures for each variant. As shown (Figures 1C,D), the RNA structure-analysis algorithm predicted the formation of a hairpin encompassing the miR-23ab binding site, shown with an estimation of base-pairing probability. The minimum free energy of the thermodynamic ensemble was −4.80 Kcal/mol for the most common T variant vs. −7.30 Kcal/mol for the C variant, indicating greater stability for the latter. This observation agrees with the calculated positional-entropy for each structure (Figure 1E). The minimum free energies for miR-23-mRNA dimer formation under both circumstances were calculated with RNAcofold (34), revealing predicted values of −13.95 Kcal/mol for the T allele and −16.13 Kcal/mol for the C variant allele, meaning that heterodimer formation with miR-23 was also favored by the rs17735770 variant.

### Functional Evaluation of miR23 Binding

**Figure 2:**
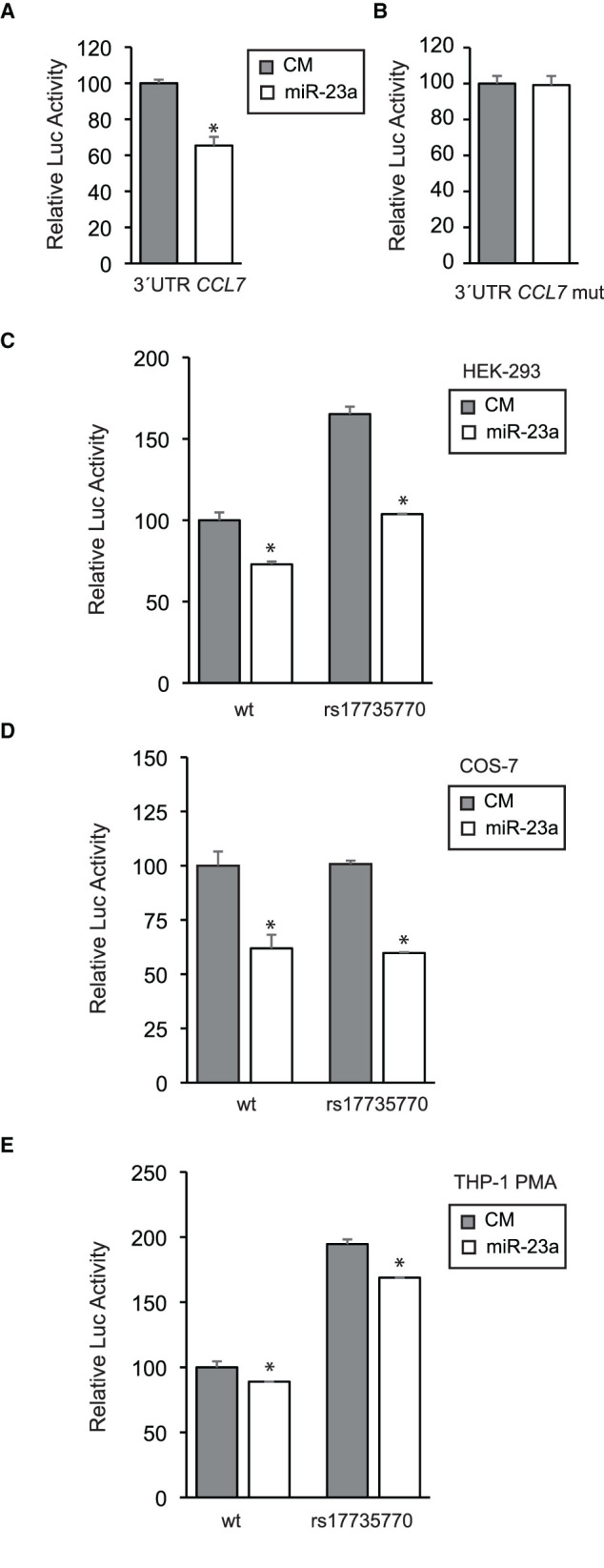
Functional evaluation of the miR-23 binding site at the human CCL7 3'UTR. **(A)** The human CCL7 gene 3'UTR was cloned downstream of a Renilla luciferase reporter and transfected into HEK-293 cells along with a miR-23a-3p mimic or a nontargeting control mimic (CM) as indicated. After 24 h, *Renilla* luciferase activity was measured and normalized according to Firefly luciferase activity, present in the same construct under a housekeeping promoter and a SV40 polyadenylation signal. The relative luciferase activity of the control was assigned to 100% activity as shown. **(B)** Alternatively, the miR-23 consensus site on the CCL7 gene 3'UTR was mutated from AATGTGAA to AATCAGGA and tested as above. **(C)** The rs17735770 variant was introduced into the CCL7 gene 3'UTR and cloned downstream of a *Renilla* luciferase reporter as the wt counterpart. Both the wt and rs17735770 variant versions were transfected into HEK-293 cells and tested as above in the presence of CM or miR-23a-3p mimics as indicated. A similar experimental approach was followed in COS-7 **(D)** and THP-1 cells treated with 100 nM PMA 24 h before transfections **(E)**. Data are expressed as relative luciferase activities compared to the activity in control samples assigned to 100% activity co-transfected with an equal concentration of the CM and correspond to the means ± SEM from three experiments performed in triplicate. * *P* < 0.05 (significantly different from cells co-transfected with CM and the WT or mut 3′ UTR) ***p ≤ 0.001.

The functional relevance of the putative miR-23 binding site at the human *CCL7* 3'UTR was evaluated in the context of heterologous cellular assays. Firstly, synthetic DNA plasmid constructs containing the human *CCL7* 3'UTR downstream of the coding sequences of the *Renilla* luciferase gene were transfected into human embryonic kidney HEK-293 cells along with either miR-23a-3p mimic or nontargeting control mimic (CM) sequence As shown, overexpression of miR-23 significantly repressed *CCL7* 3'UTR activity (Figure 2A), while the mutation of the miR-23 binding site relieved the repression, which is consistent with its direct interaction with the human *CCL7* 3'UTR (Figure 2B) (40).

Once the miR-23 binding site was validated, similar analysis were performed to compare constructs containing either the common (wild type) or the human *CCL7* 3'UTR containing the rs17735770 variant. As shown, the construct containing the variant showed higher activity than the wt counterpart while inhibitory effect of miR-23a on the 3'UTR remained intact (Figure 2C).

To further explore this effect in other cell lines, COS-7 transformed African green monkey kidney fibroblasts were subjected to transfection with either common or variant 3'UTR along with CM or miR-23a mimic as indicated (Figure 2D). These analyses revealed that the levels of expression of the *Renilla* luciferase reporter under either the wild type or the variant *CCL7* 3'UTR were similar, and both were inhibited upon co-transfection with miR-23a.

Finally, we further studied *CCL7* 3'UTR activity in a native CCL7-expressing background, the human monocyte THP-1 cell line differentiated toward macrophage-like cells by addition of 100 nM PMA (41), leading to increased endogenous CCL7 expression (42). In this setting, the 3'UTR activity containing either the common or the variant were both significantly repressed by miR-23a and, in agreement with the results obtained in HEK-293 cells, a higher basal activity was detected for the construct containing the variant *CCL7* 3'UTR downstream of the reporter gene (Figure 2E).

## Discussion

Atherosclerosis is the result of the chronification of an inflammatory process that fails to resolve and where monocytes play a key role from start. While much is known about the mechanisms that participate in the initiation and evolution of the disease, much less is known about the signals that may lead to its resolution (43). In this study, we have approached the study of classical risk factors associated with coronary heart disease in combination with genetic variants in genes that regulate monocyte function, in particular the CCR2 receptor and its ligands.

The characteristics of the population of patients presented in this study are similar to other populations and, accordingly, associated with known risk factors (44, 45). Elderly males, smokers, and patients presenting with arterial hypertension, diabetes or dyslipidemia were significantly associated with cardiovascular disease when compared to the control set. It was interesting to observe how specific risk factors segregated differentially according to the type of event suffered. While dyslipidemia and arterial hypertension were more prevalent among patients with angina pectoris, current smoking status and elevated inflammatory markers, including total leukocyte and monocyte counts, were more likely associated with acute coronary events in younger patients. In fact, cigarette smoking is the most common and modifiable risk factor in young adult patients with myocardial infarction (46) whereas diabetes and hypertension are less frequent (47). Also, single vessel coronary disease is more common than three vessel diseases in younger patients, as they are exposed to fewer cardiovascular risk factors, leading to reduced collateral circulation, a greater frequency of STEMI (48) and a higher myocardial area at risk ultimately leading to myocyte necrosis and to an increase in inflammatory markers.

A multivariant analysis further supported strong independent associations of age, smoking status, arterial hypertension, and dyslipidemia with cardiovascular risk in this population. It is well established that there is a genetic heritage behind some of these factors and, possibly, other physiological mechanisms that may also influence the susceptibility to suffer chronic diseases (49). It could be expected that specific genetic traits would become evident before age-dependent or other environmental factors exert their effect (50). Therefore, there might be a need to exclude general risk factors in order to detect genetic differences that could be risk factors *per se*. So, to approach this analysis, cases and controls were matched considering all the variables that were identified to be associated with the disease phenotype through a multivariant logistic regression analysis, generating a set of homogeneous pairs of cases and controls with similar characteristics.

The CCR2 receptor and its ligands were selected as candidate genes for a genetic study because of their importance in macrophage biology and because both agonist and antagonist functions are known for this setting, providing a window of opportunity for a negative feedback control mechanism that could lead to inflammation resolution (16). CCR2 is necessary for the development of atherosclerosis, as it has been demonstrated in murine models of atherosclerosis where either the *CCR2* gene or the *CCL2* gene, encoding for one of its ligands, are inactivated (11–15). Although later work reveals a key role of both CCR1 and CCR5 in monocyte recruitment leading to atherogenesis (51), it is also evident that, beyond facilitating the egress of monocytes from the bone marrow to increase their circulating numbers (9, 10, 52), CCR2 and its ligands are also autonomously involved in remodeling the endothelium in inflammatory processes and in the mobilization of monocytes associated with it (53–55).

From all *CCR2, CCL2* and *CCL7* variants identified, only rs17735770, located at the 3'end of the *CCL7* gene, was found to segregate preferentially with the absence of cardiovascular disease, an observation that was further substantiated when analyzed in a greater case-control-matched population, where all pairs shared identical risk factors. This group was represented mostly by men with an average age of 57, indicating that this genetic variable influenced or modified disease risk before age-related factors take place.

The association of this variant, at the 3'end of the *CCL7* gene, with the healthy phenotype is of interest and, accordingly, since the propensity score approach used in this study eliminates differences in other risk factors, setting a background to detect genetic modifiers, it has not been previously linked with cardiovascular disease, as suggested by GWAS exploring the association of genetic variants with a variety of cardiovascular disease-associated traits (56). This variant was predicted to alter the local RNA structure (Figure 1), lied close to a binding site for miR-23ab, and its presence resulted in increased stability of both an internal hairpin and miR-23ab dimer formation, and also in enhanced expression of a reporter in a heterologous assay, regardless of exogenous miR-23a addition.

Single nucleotide polymorphisms in regulatory non-coding RNAs such as miRNAs, as well as their direct binding sites or their vicinity at the 3‘UTR regions have been previously associated with human disease (57). Indeed, similar to our observations in the CCL7 3'UTR, a variant closely located to a miR-199a binding site at the 3'UTR of the HIF1A gene, is associated with pancreatic ductal adenocarcinoma risk and worse clinical outcomes (58). In this case, however, the rs17735770 variant did not hamper miR-23 binding, but rather resulted in increased CCL7 3'UTR activity independently of the participation of miR-23 in a heterologous assay in two different cell lines. These results suggest that variation resulting from rs17735770 might interfere with additional factors that regulate CCL7 expression at the posttranscriptional level. Given the function of miRNAs in the control of mRNA translation and stability (59), it is tempting to speculate that the presence of the rs17735770 variant results in increased expression of CCL7 at the sites of inflammation, where it could become an antagonist and ultimately contribute to the resolution of inflammation.

Little is known about the association of these miRNAs with cardiovascular disease and/or inflammation. Increased miR-23 levels are associated with the maintenance of the integrity of endothelial structures (60), and has been proposed to evaluate the presence and severity of coronary lesions in CHD patients (61) while, in an experimental autoinmmune murine model of encephalomyelitis, miR-23b suppresses leukocyte migration and pathogenesis by targeting CCL7 (39).

Our findings invite to revisit the experimentation on therapeutic approaches based on CCR2 antagonists for the prevention of cardiovascular disease. Because their influence in inflammation and monocyte biology, chemokines and their receptors have been the subject of pharmaceutical targeting for atherosclerosis and related conditions in recent years (62). In murine preclinical models of atherosclerosis, both directed antagonism to either CCR2 or a subset of chemokine receptors, including CCR2, result in a reduction in plaque formation (63, 64). However, clinical trials in humans have not been very informative in this area. A beneficial reduction of serum C Reactive Protein (CRP) has been recorded in patients treated with MLN1202, a humanized monoclonal antibody disrupting the interaction of CCR2 with CCL2 and, likely, with other ligands as well (65). These effects, accompanied by an overall reduction in circulating monocytes, are seen after a systemic exposure to MLN1202 in patients that have a promoter genetic variant at the *CCL2* gene which results in increased CCL2 expression. In this setting, antagonism at CCR2 not only blocks monocyte migration into inflammatory lesions but also their release into circulation, also mediated by the CCR2-CCL2 interaction (6), and could lead to unwanted side effects derived from monocyte retrieval from circulation.

The impact of increased expression of CCL7 at inflammatory sites, and its function as an antagonist, as we speculate from our results, supports the idea that antagonism directed at the plaque should cause local benefit without compromising innate immunity elsewhere. Indeed, selective targeting of anti-inflammatory microRNAs to inflamed endothelium in a mouse model of atherosclerosis, lead to a local reduction in the expression of CCL2, among other chemokines, accompanied by reduced inflammation, macrophage numbers and plaque size (66). Recent observations revealing a circadian nature for the CCR2-CCL2 interaction at the plaque open the possibility for specific pharmaceutical intervention when inflammatory activity at the plaque is higher, a timely fashion to achieve selective targeting (67).

We acknowledge that further experimental evidence will be necessary to give a solid functional significance to the rs17735770 genetic variant as cardioprotective. The benefit of increased local CCL7 expression at the plaque and a differential expression between carriers and non-carriers of the rs17735770 variant is an issue that is difficult to approach in humans, but may inspire the design of novel therapeutic approaches.

## Data Availability Statement

The original contributions presented in the study are included in the article/Supplementary Materials, further inquiries can be directed to the corresponding author.

## Ethics Statement

The studies involving human participants were reviewed and approved by CEIm PROVINCIAL LAS PALMAS Barranco de la Ballena, s/n Edificio Anexo al Hospital Universitario de Gran Canaria Dr. Negrín 35019-Las Palmas de Gran Canaria, Spain, email: ceimprovlpa.scs@gobiernodecanarias.org. The patients/participants provided their written informed consent to participate in this study.

## Author Contributions

JM-G, EM-Q, and FR-G collected, processed, and analyzed patient information. AP-G and CR designed and performed miRNA functional studies. PS-S was performed the statistical analysis. AD-C, PG-S, and AT performed sequencing and genotyping. MR supervised biochemical laboratory work. AT conceived the work, participated in data collection and analysis, prepared the figures and wrote the manuscript, which was finally supervised, modified, and finally approved by all authors.

## Funding

This project has been initially funded by FUNCIS Project (PI 16/06) to EM-Q, by the Servicio Canario de Salud, and by grants RTI2018-095061-B-I00 (Convocatoria 2018 de proyectos de I+D+i ≪RETOS INVESTIGACIÓN≫) from the Ministerio de Ciencia, Investigación y Universidades, and TALENTO Grant 2017-T1/BMD-5333 from Comunidad de Madrid both to CR. AP-G is employed through PEDJ-2018-POST/BDM-8900 (Ayudas para la contratación de investigadores postdoctorales) from Consejería de Educación e Investigación from the Madrid Government, Spain, awarded to CR and AP-G.

## Conflict of Interest

AD-C was employed by SECUGEN SL, Madrid, Spain and designed all the primers and performed all the sequencing associated with this project at SECUGEN SL, a service paid for by the authors. However, SECUGEN SL was not involved in the study design, collection, analysis, interpretation of data, the writing of this article or the decision to submit it for publication. The remaining authors declare that the research was conducted in the absence of any commercial or financial relationships that could be construed as a potential conflict of interest.

## Publisher's Note

All claims expressed in this article are solely those of the authors and do not necessarily represent those of their affiliated organizations, or those of the publisher, the editors and the reviewers. Any product that may be evaluated in this article, or claim that may be made by its manufacturer, is not guaranteed or endorsed by the publisher.
