# Supplementary material for: A Common Variant at the 3'untranslated Region of the CCL7 Gene (rs17735770) Is Associated With Decreased Susceptibility to Coronary Heart Disease
Source: Front Cardiovasc Med. 2022 May 31;9:908070. doi: 10.3389/fcvm.2022.908070 (PMC9194478; doi:10.3389/fcvm.2022.908070)
Supplement: Supplementary file 1 [file Table_1.pdf]

Supplementary Table 1.

Gene	Position [a]	Alleles [b]	SNP ID [c]	Region [d]	Protein variant [e]	Freq gnomAD [f]	Cases (N=70) [g]			Controls (N=30)			p
							Ref	Het	Hom	Ref	Het	Hom	
CCR2	3:46353822	A/G	rs3749461	Exon1 - 5´UTR		G=0.056	46	7	0	14	6	1	0,071
	3:46354020	-/T	rs36225401			ND	52	16	1	21	8	0	0,739
	3:46354094	G/A	rs3092964			G=0.2	4	29	16	1	7	13	0,07
	3:46354124	T/C	rs3918376			C=0.0046	66	3	0	28	1	0	0,837
	3:46354176	T/C	novel			ND	48	1	0	21	0	0	0,510
	3:46354213	T/C	novel			ND	68	1	0	29	0	0	0,515
	3:46354226	G/T	rs140800049	Intron		T=0.0007	49	1	0	21	0	0	0,514
	3:46354295	G/A	rs3918361			A=0.2	1	48	4	0	20	0	0,363
	3:46357683	G/T	rs3918367	Exon 2 - ORF	NP_001116513.2:p.V52	T=0.007	69	1	0	29	0	0	0,518
	3:46357717	G/A	rs1799864		NP_001116513.2:p.V64I	A=0.1212	61	8	1	22	7	0	0,233
	3:46359752	A/G	rs3918386		NP_001116513.2:p.P339	G=0.06	65	5	0	29	0	0	0,140
	3:46360039	--C/TGT	rs372263390	Exon 2 - 3´UTR		ND	69	1	0	29	0	0	0,518
	3:46360115	A/G	rs140253702			G=0.009	68	1	0	28	1	0	0,523
	3:46360527	G/A	rs743660			A=0.2	23	44	3	18	10	1	0,026
	3:46360562	T/G	rs34138562			G=0.13	62	8	0	22	7	0	0,108
	3:46360940	A/T	rs11575062	Downstream		T=0.057	61	9	0	21	7	1	0,102
CCL2	17:34256250	T/C	rs4586	ORF	NP_002973.1:p.C35	T=0.4742	23	44	3	18	9	3	0,015
	17:34256892	C/T	rs13900	3´UTR		T=0.2868	43	26	1	22	6	1	0,249
CCL7	17:34271039	C/T	rs9901581	Intron		T=0.056	69	1	0	29	0	0	0,525
	17:34271319	G/A	rs70937036			A=0.0043	68	2	0	29	0	0	0,366
	17:34271356	A/G	rs16969398			G=0.0082	70	0	0	27	1	0	0,112
	17:34271923	T/C	rs17735770	3´UTR		C=0.037	68	2	0	23	6	0	0,003

Supplementary Table. Genetic variants for the CCR2, CCL2 and CCL7 genes in cases and controls. Exons and flanking sequences for the CCR2, CCL2 and CCL7 genes were subjected to double-pass heterozygous Sanger sequencing in 70 cases and 30 controls. **(a)** Position of the variant according to reference genome GRCh38 (chromosome: position). **(b)** Alleles found for each variant (reference/variant) **(c)** SNP ID in dbSNP (built 134/151). **(d)** Region of the gene where the variant is found; ORF: Open Reading Frame; UTR: Untranslated Region; Downstream: Beyond the 3´end. **(e)** Aminoacid changes according to the reference peptide. **(f)** Frequency of the variant allele at the gnomAD database (https://gnomad.broadinstitute.org/). **(g)** Total count of individuals found in cases and controls that were either wt (no variant; Ref), heterozygous (Het) or homozygous (Hom) for the variant or least frequent allele.
