# Supplementary material for: A Common Variant at the 3'untranslated Region of the CCL7 Gene (rs17735770) Is Associated With Decreased Susceptibility to Coronary Heart Disease
Source: Front Cardiovasc Med. 2022 May 31;9:908070. doi: 10.3389/fcvm.2022.908070 (PMC9194478; doi:10.3389/fcvm.2022.908070)
Supplement: Supplementary file 2 [file Data_Sheet_1.DOCX]

**SUPPLEMENTARY METHODS. PRIMER SEQUENCES.**

The name of the primer is shown, followed by the sequence (5´to 3´)

**Amplification and sequencing coding sequences of the human CCR2, CCL2 and CCL7 genes.**

CCR2-UTR-F GGAACATGAAACATTCTGTTGTGC;

CCR2-UTR-R CCCTGCCTCTCATCTTGATC

CCR2-Ex1-1F GATGTCTGCCACTAGGAATGAAAG

CCR2-Ex1-1R TATCGATTGTCAGGAGGATGATG

CCR2-Ex1-2F CATTGTGGGCTCACTCTGCTGC

CCR2-Ex1-2R TTGGATATGCATTGGGTGACATAG

CCR2-Ex2-1F AATGATGAGTTCCTTCACCAGGAG

CCR2-Ex2-1R CCACATTTACAAGTTGCAGTTTTCAGC

CCR2-Ex2-2F CAGTCTTCAGGACAAAGAAGGAGC

CCR2-Ex2-2R TTTGCTTACTTCAGGGAGCTTCTC

CCR2-Ex2-3F GAATCACAGTATACGCTCCATCGC

CCR2-Ex2-3R GCACTGCAAGTCACACCACAAG

CCL2-Ex1-F TTCCTACTTCCTGGAAATCCACAG

CCL2-Ex1-R CTGGAATTCTAGTTCAGGCTCTGC

CCL2-Ex2-F CTGAGGTATAGGCAGAAGCACTG

CCL2-Ex2-R CCATTGGGAACCTTTGTACATTAAG

CCL2-Ex3-F TGGACACCTATAGGAGCAGTTTGC

CCL2-Ex3-R CCCTCTGAAATCATGTTCCTTTGTC

CCL7-Ex1-F AGACCCTACCTCACCTACCACTCC

CCL7-Ex1-R CTGGAATTCTAGTTCAGGCTCTAC

CCL7-Ex2-F CTCTTCCGTCTTTCAACTGGTGATG

CCL7-Ex2-R AAATGATAGCTGGAAAGGGAGCTG

CCL7-Ex3-F ATCTCAGTTCGTTCCTTCATCCTG

CCL7-Ex3-R AGTCCATTACGCGCTCATTTCTC

**RFLP assay for rs17735770**

CCL7_B1_1F CCCAGGCTGAACCCTCAAGGTG

CCL7_B1_1R AGAAGGGAGGAGCATCCCACAGT

**Cloning of human CCL7 gene 3’UTR**

hCCL7_fw CCCctcgagGCACCTGGACAAGAAAACCC

hCCL7_rv CCCgcggccgcTTTATGTGAAAATTTGGGAGTC

**Mutagenesis**

hCCL7pmSNIP_fw ctaagaaaaaccatgattcacattattacgtaaagctcatattccttagaattagatta

hCCL7pmSNIP_rv taatctaattctaaggaatatgagctttacgtaataatgtgaatcatggtttttcttag-3'

hCCL7pm8MER_fw aatctactaagaaaaaccatgattctgattattacataaagctcatattccttagaattagatt

hCCL7pm8MER_rv aatctaattctaaggaatatgagctttatgtaataatcagaatcatggtttttcttagtagatt
